# Complex Pattern of Resistance-Associated Substitutions of Hepatitis C Virus after Daclatasvir/Asunaprevir Treatment Failure

**DOI:** 10.1371/journal.pone.0165339

**Published:** 2016-10-24

**Authors:** Jun Itakura, Masayuki Kurosaki, Chitomi Hasebe, Yukio Osaki, Kouji Joko, Hitoshi Yagisawa, Shinya Sakita, Hiroaki Okushin, Takashi Satou, Hiroyuki Hisai, Takehiko Abe, Keiji Tsuji, Takashi Tamada, Haruhiko Kobashi, Akeri Mitsuda, Yasushi Ide, Chikara Ogawa, Syotaro Tsuruta, Kouichi Takaguchi, Miyako Murakawa, Yasuhiro Asahina, Nobuyuki Enomoto, Namiki Izumi

**Affiliations:** 1 Department of Gastroenterology and Hepatology, Musashino Red Cross Hospital, Musashino, Tokyo, Japan; 2 Department of Gastroenterology, Japanese Red Cross Asahikawa Hospital, Asahikawa, Hokkaido, Japan; 3 Department of Gastroenterology and Hepatology, Osaka Red Cross Hospital, Osaka, Osaka, Japan; 4 Center for Liver-Biliary-Pancreatic Disease, Matsuyama Red Cross Hospital, Matsuyama, Ehime, Japan; 5 Department of Gastroenterology, Japanese Red Cross Akita Hospital, Akita, Akita, Japan; 6 Department of Gastroenterology, Yokohama City Minato Red Cross Hospital, Yokohama, Kanagawa, Japan; 7 Department of Gastroenterology, Japanese Red Cross Society Himeji Hospital, Himeji, Hyogo, Japan; 8 Department of Gastroenterology, Nasu Red Cross Hospital, Otawara, Tochigi, Japan; 9 Department of Gastroenterology, Date Red Cross Hospital, Date, Hokkaido, Japan; 10 Department of Gastroenterology, Maebashi Red Cross Hospital, Maebashi, Gunma, Japan; 11 Department of Gastroenterology, Hiroshima Red Cross Hospital & Atomic-bomb Survivors Hospital, Hiroshima, Hiroshima, Japan; 12 Department of Gastroenterology, Takatsuki Red Cross Hospital, Takatsuki, Osaka, Japan; 13 Department of Gastroenterology, Japanese Red Cross Okayama Hospital, Okayama, Okayama, Japan; 14 Department of Gastroenterology, Japanese Red Cross Tottori Hospital, Tottori, Tottori, Japan; 15 Department of Gastroenterology, Japanese Red Cross Karatsu Hospital, Karatsu, Saga, Japan; 16 Department of Gastroenterology, Takamatsu Red Cross Hospital, Takamatsu, Kagawa, Japan; 17 Department of Gastroenterology, Japanese Red Cross Nagasaki Genbaku Hospital, Nagasaki, Nagasaki, Japan; 18 Department of Hepatology, Kagawa Prefectural Central Hospital, Takamatsu, Kagawa, Japan; 19 Department of Gastroenterology and Hepatology, Tokyo Medical and Dental University, Bunkyo, Tokyo, Japan; 20 First Department of Internal Medicine, University of Yamanashi, Chuou, Yamanashi, Japan; National Taiwan University Hospital, TAIWAN

## Abstract

**Backgrounds & Aims:**

We aimed to clarify the characteristics of resistance-associated substitutions (RASs) after treatment failure with NS5A inhibitor, daclatasvir (DCV) in combination with NS3/4A inhibitor, asunaprevir (ASV), in patients with chronic hepatitis C virus genotype 1b infection.

**Methods:**

This is a nationwide multicenter study conducted by the Japanese Red Cross Liver Study Group. The sera were obtained from 68 patients with virological failure after 24 weeks of DCV/ASV treatment. RASs in NS5A and NS3 were determined by population sequencing.

**Results:**

The frequency of signature RASs at position D168 of NS3 was 68%, and at positions L31 and Y93 of NS5A was 79 and 76%, respectively. The frequency of dual signature RASs in NS5A (L31-RAS and Y93-RAS) was 63%. RASs at L28, R30, P32, Q54, P58, and A92 in addition to dual signature RAS were detected in 5, 5, 1, 22, 2, and 0 patients, respectively. In total, triple, quadruple, and quintuple RASs in combination with dual signature RAS were detected in 35, 10, and 1.5% patients, respectively. These RASs were detected in patients without baseline RASs or who prematurely discontinued therapy. Co-existence of D168 RAS in NS3 and L31 and/or Y93 RAS in NS5A was observed in 62% of patients.

**Conclusion:**

Treatment-emergent RASs after failure with DCV/ASV combination therapy are highly complex in more than 50% of the patients. The identification of complex RAS patterns, which may indicate high levels of resistance to NS5A inhibitors, highlights the need for RAS sequencing when considering re-treatment with regimens including NS5A inhibitors.

## Introduction

The treatment of chronic hepatitis C virus (HCV) infection has evolved rapidly in recent years. The practical application of direct-acting antivirals (DAAs) is the driving force behind this progress. Currently, treatments comprising DAAs only, without incorporation of interferons, have been developed. For HCV genotype 1, 24-week combination therapy with daclatasvir (DCV) and asunaprevir (ASV) was the first all-oral DAA regimen to be licensed in Japan [1]. DCV is a potent, first-in-class, NS5A replication complex inhibitor with pan-genotypic activity [2]. ASV is a NS3 protease inhibitor [3]. A Japanese phase 3 study reported proportions of patients achieving sustained virological response (SVR) after DCV/ASV combination therapy in 87.4% of interferon-ineligible/intolerant patients and 80.5% of non-responders to prior therapy.

In HCV genome, the resistance-associated substitutions (RASs) present at specific sites confer resistance to DAAs [4,5]. According to *in vitro* genotype 1b replicon assays, the signature D168V RAS in NS3 confers 280-fold resistance to ASV compared to D168-wild type [6]. Among the various RASs in NS5A, L31 and Y93 represent the signature RASs against DCV in HCV genotype 1b [5,7–9]. *In vitro* genotype 1b replicon assays have demonstrated that L31V, Y93H, and dual RASs of L31V plus Y93H confer 28-, 24-, and >14,000-fold resistance to DCV, respectively. In is noteworthy that single RAS may be observed in DAA-naive patients to some extent [10–14]; however, dual RASs are extremely rare [15,16]. Baseline RASs attenuates the efficacy of DCV/ASV [17,18]. Moreover, treatment failure induces the emergence of highly resistant RASs [4,19,20]. In addition, many of the RASs in NS5A may be cross-resistant to other DAAs of the same class [21,22].

Recently developed regimens have demonstrated efficacy in DAA-naive patients with baseline RASs in NS5A [23–25]; however, their efficacy for the re-treatment of NS5A inhibitor-experienced patients remains unclear. Current guidelines postulate that the efficacy of re-treatment may be dependent on the treatment-emergent RASs, and therefore, American Association for the Study of Liver Diseases/ Infectious Diseases Society of America (AASLD/IDSA) [26] and The Japan Society of Hepatology (JSH) guidelines [27] recommend testing for treatment-emergent RASs. Therefore, in this study, we aimed to identify the characteristics of RASs after treatment failure using DCV/ASV combination therapy in patients with HCV 1b infection.

## Materials and Methods

### Patients

Serum was collected from the 68 patients who failed to achieve SVR by DCV/ASV combination therapy (DCV 60 mg daily, ASV 100 mg twice daily, 24weeks) carried out in the 96 institutes participating in the Japanese Red Cross Liver Study Group. Reasons for treatment failure were as follows: virological breakthrough with transient disappearance of serum HCV RNA during therapy (n = 36), relapse after end of therapy (n = 17), non-response with continued detection of serum HCV RNA during therapy (n = 5), and stopped therapy by adverse events (n = 10). The median duration of treatment was 18 weeks. Serum samples were collected as soon as possible after treatment failure. The median duration between the end of treatment and serum collection was 15.5 weeks (0–93 weeks).

Table 1 presents clinical background of patients. Median serum HCV RNA were 6.2logIU/ml with range from 3.5logIU/ml to 7.2logIU/ml. About 22% of patients experienced prior therapy with DAAs, telaprevir or simeprevir, and about 21% had the history of previous treatment of hepatocellular carcinoma. All but except 1 patient measured baseline L31 and Y93 RASs in NS5A before DCV/ASV therapy. About 10% and 25% of patients had L31 and Y93 RASs, and only 3 patients (4.4%) had dual RASs (L31-RAS/Y93-RAS) before DCV/ASV therapy. Other RASs in NS3 and in NS5A at baseline was not examined.

**Table 1:** Baseline characteristics.

	n = 68
Age (years)	68.2 ± 8.8
Male (%)	35.3
AST (U/L)	61.2 ± 35
ALT (U/L)	53.2 ± 33
Platelet count (×10^−9^/L)	128 ± 8.2
Platelet count <100 × 10^−9^/L, %	32.3
Albumin (g/dl)	3.87 ± 0.38
AFP (ng/ml)	27.5 ± 49
HCV RNA (RT-PCR, logIU/ml)	6.14 ± 0.61
Previous treatment contents	
(naive or IFN monotherapy/ PR therapy/ PR+TVR/ PR+SMV, %)	34.3/ 43.2/ 7.5/ 15.0
Prior treatment of HCC (yes, %)	20.9
RASs at baseline (NS5A)	
L31 (wild/ variant/ not examined, %)	88.2/ 10.3/ 1.5
Y93 (wild/ variant/ not examined, %)	73.5/ 25.0/ 1.5
Outcome of DCV/ASV combination therapy	
(stopped by AE/ Breakthrough/ Relapse/ No response, %)	13.4/ 53.7/ 25.4/ 7.5
Duration of DCV/ASV therapy	
(≤12 weeks/ 13–23 weeks/ 24 weeks, %)	32.8/ 29.9/ 37.3

AE, adverse event’ AFP, alpha-fetoprotein’ ALT, alanine aminotransferase’AST, aspartate aminotransferase’ ASV, asunaprevir’ DAA, direct-acting antivirals’ DCV, daclatasvir’ HCC, hepatocellular carcinoma’ IFN, interferon’ PR, pegylated interferon + ribavirin’ RAS, resistance-associated substitution’ RBV, ribavirin’ SMV, simeprevir’ TVR, telaprevir’

Written informed consent was obtained from each patient, and we conformed to the ethical guidelines of the Declaration of Helsinki. This study was approved by the institutional ethics review committee of Musashino Red Cross Hospital (approval number 499).

### Analysis by direct sequencing

Direct sequencing was used to detect RASs in NS3 and NS5A regions, as described previously [16]. Briefly, viral RNA was extracted from serum using QIAamp Viral RNA Mini Kits (QIAGEN). The extracted RNA was reverse-transcribed and amplified by the two-step nested PCR method using the SuperScript III One-Step RT-PCR System with Platinum Taq DNA Polymerase (Invitrogen), with specific pairs of primers. The PCR products were purified using QIAquick PCR Purification Kit (QIAGEN) and sequenced using an automated DNA sequencer (3730xl DNA Analyzer, Applied Biosystems). Each sequence was confirmed for both sense and anti-sense strands. Sequences with codon changes detected in more than 10% of the total sequence were regarded as positive.

For analysis, predicted HCV amino acid sequences from patients were compared with the sequence of the HCV-J strain (GenBank Accession No.AJ238799, http://www.ncbi.nlm.nih.gov/nuccore/AJ238799.1) as a reference. RASs in NS5A (L28, R30, L31, P32, Q54, P58, A92, and Y93) and in NS3 (T54, Q80, S122, R155, A156, and D168) were determined [5,8,28,29].

### Statistical analysis

P values < 0.05 were considered statistically significant. All statistical analyses were performed with EZR (Saitama Medical Center, Jichi Medical University, Saitama, Japan), which is a graphical user interface for R (The R Foundation for Statistical Computing, Vienna, Austria). More precisely, it is a modified version of R commander designed to add frequently used statistical functions [30].

## Results

### Overview of the prevalence of RASs after daclatasvir/asunaprevir failure

**Fig 1:**
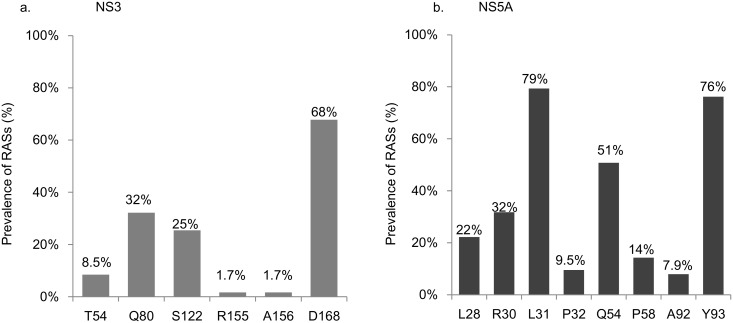
Prevalence of RASs after failure of combination therapy with daclatasvir and asunaprevir. Prevalence of RAS in NS3 (a) and NS5A(b).

Fig 1 shows the frequency of RASs. Table 2 shows the patterns of RAS after DCV/ASV failure. Sequences in the NS3 region were successfully amplified and determined in 59 patients. The frequencies of the RASs in NS3 region were as follows: 1.7% for R155 and A156, 8.5% for T54, 32% for Q80 (Q80K, 26%; Q80L, 32%; Q80R, 53%), 25% for S122 (S122G, 73%; S122I, 20%; S122T, 20%), and 68% for D168 (D168E, 63%; D168V, 20%, other RASs, 30%). Twelve out of 59 patients (20%) had no RASs. Sequences in the NS5A region were successfully amplified and determined in 63 patients. The frequencies of RASs in NS5A region were as follows: 76% for Y93 (H, 98%; R, 2%) and 79% for L31 (L31F, 24%; L31I, 24%; L31M, 38%; L31V, 38%). In addition to these signature RASs, the frequencies of RAS at L28, R30, P32, Q54, P58, and A92 in NS5A were 22% for L28, 32% for R30, 9.5% for P32 (P32 deletion, 50%), 51% for Q54, 14% for P58, and 7.9% for A92.

**Table 2:** RAS in NS3 and NS5A regions after failure of daclatasvir/asenaprevir combination therapy.

NS3	Cases	NS5A	Cases
T54S	1	L28I+R30H	1
T54S+D168E	1	L28M/T+R30Q+L31F+A92K	1
T54S+D168E/K	1	L28M+Q54Y+Y93H	1
T54S+Q80L+R155K	1	L28M+R30H/Q+L31I/V+Y93H	1
T54S+S122G+A156S+D168E	1	L28M+R30H+L31F	1
Q80K	1	L28M+R30L+L31V+P32L	1
Q80K/R+D168E	2	L28M+R30Q+L31F+Y93R	1
Q80K+D168E	2	L28M+R30Q+L31I+Y93H	1
Q80L	1	L28M+R30Q+L31M+Y93H	1
Q80L+D168E	1	L28M+R30Q+L31V+A92K	1
Q80L+D168E/V	1	L28M+R30Q+Y93H	1
Q80L+S122G	1	L28S+L31V+P58S+A92K	1
Q80L+S122T+D168A/T	1	L28T+R30H+Q54H	1
Q80R	1	L28V+R30H/Q+L31F+Q54L+Y93H	1
Q80R+D168E	6	R30H+P32L+Q54H	1
Q80R+S122I+D168E	1	R30H+P58K+Y93H	1
S122G	1	R30H+Y93H	1
S122G+D168E	2	R30Q+L31F+Q54H	1
S122G+D168H	1	R30Q+L31F+Y93H	1
S122G+D168N/S/T/Y	1	R30Q+L31I/M/V+Y93H	1
S122G+D168T	2	R30Q+L31M+Y93H	1
S122G+D168V	2	R30Q+Q54H+A92K	1
S122I/T+D168V	1	L31F/I/M+Q54H+Y93H	1
S122T+D168E	1	L31F/I/V+Q54H+Y93H	1
D168A	1	L31F/V+P58Q+Y93H	1
D168E	6	L31F+P32del	1
D168T	1	L31F+P32del+Q54H+P58A/T	1
D168V	4	L31F+Q54H+Y93H	1
D168Y	1	L31I/V+Q54H+Y93H	1
Wild	12	L31I+P58S+Y93H	1
		L31I+Q54H+P58S+Y93H	2
		L31I+Q54H+Y93H	1
		L31I+Y93H	2
		L31M/V+P32L+Y93H	1
		L31M/V+Q54H+Y93H	2
		L31M+Q54H/Y+Y93H	1
		L31M+Q54H+A92E/K	1
		L31M+Q54H+Y93H	5
		L31M+Q54L+Y93H	1
		L31M+Y93H	4
		L31V+Q54H+P58L+Y93H	1
		L31V+Q54H+P58S+Y93H	1
		L31V+Q54H+Y93H	4
		L31V+Y93H	2
		P32del	1
		Q54H/R	1
		Q54H+Y93H	1
		Y93H	2
Not determined	9	Not determined	5
Total	68	Total	68

### Prevalence of signature RASs in NS5A after treatment failure

**Fig 2:**
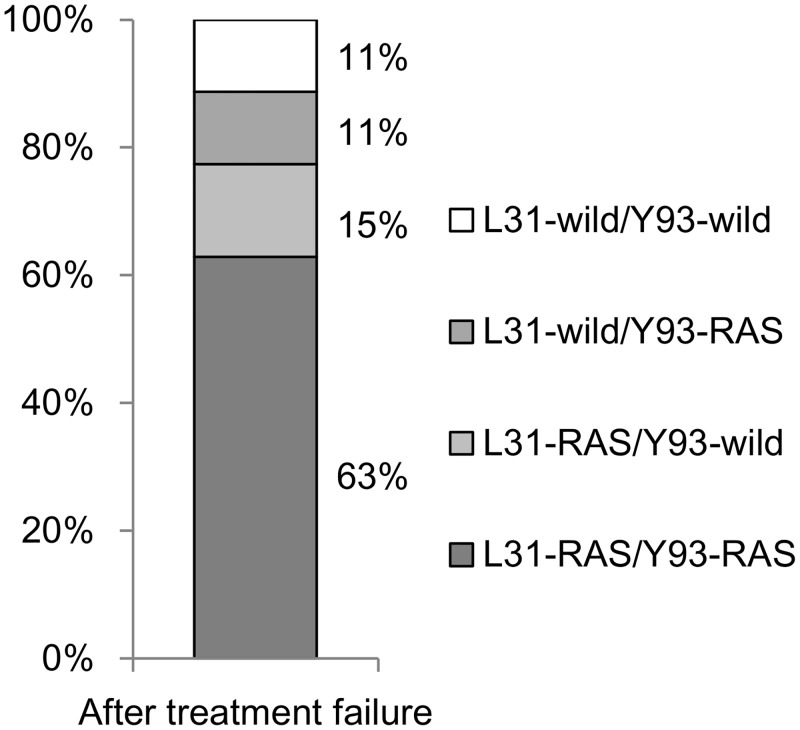
Prevalence of signature RASs in NS5A after treatment failure. The prevalence of signature RAS at L31 and Y93 in NS5A after treatment failure in total patients.

Dual RASs at L31 plus Y93 in NS5A (L31-RAS/Y93-RAS) were detected in 40 out of 63 patients (63%; Fig 2). A single RAS of L31-RAS/Y93-wild was observed in 9 (15%) patients and L31-wild/Y93-RAS in 7 (11%) patients. In total, 56 (88%) patients had either dual or single RAS at L31 and/or Y93. The prevalence of dual RASs after treatment failure was significantly higher than at baseline (3 vs. 63%; p < 0.01).

### Factors associated with dual RASs (L31-RAS/Y93-RAS) at treatment failure

**Fig 3:**
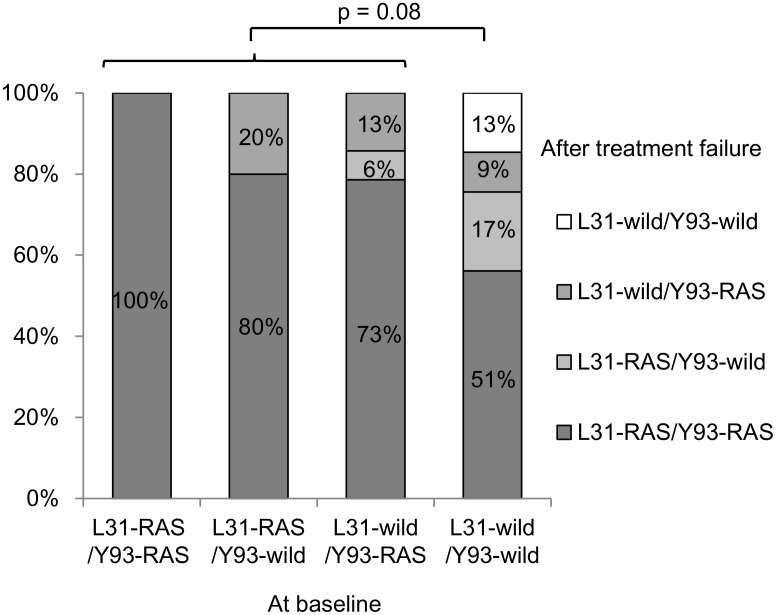
Prevalence of signature RASs in NS5A after treatment failure in terms of baseline RASs. The prevalence of signature RAS at L31 and Y93 in NS5A after treatment failure in patients stratified by presence of baseline RASs. Even in patients without L31/Y93 RAS at baseline, dual RAS at L31 plus Y93 emerged after treatment failure. The prevalence of dual RASs was not significantly different between patients with and without RASs at baseline (p = 0.08).

The prevalence of RASs at baseline and treatment-emergent RASs at treatment failure were compared (Fig 3). Treatment adherence was 100% in all patients completed the scheduled therapy. In patients who stopped therapy due to adverse events, treatment adherence was 100% during the period of treatment. The prevalence of dual RASs (L31-RAS/Y93-RAS) at treatment failure was as follows: 100% in 2 patients who had L31-RAS/Y93-RAS at baseline, 80% in patients with L31-RAS/Y93-wild at baseline, 73% in patients with L31-wild/Y93-RAS at baseline, and 51% in patients without L31 or Y93 RAS at baseline. The prevalence of dual RASs was not significantly different between patients with and without RASs at baseline (p = 0.08).

**Fig 4:**
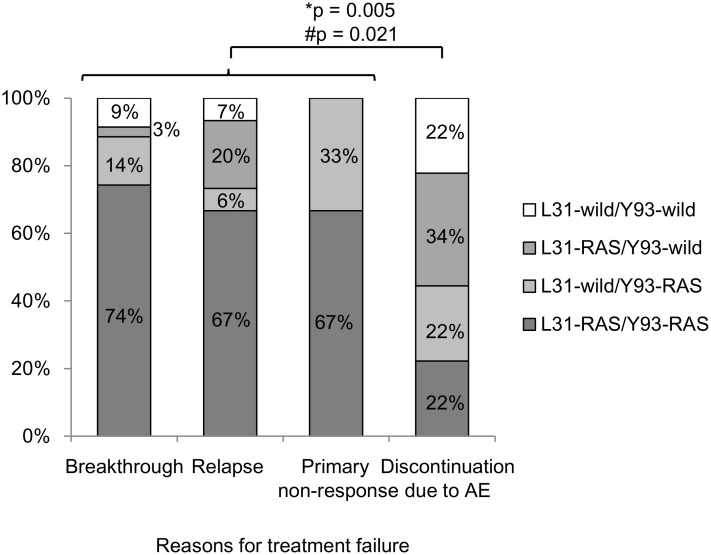
Prevalence of signature RASs in NS5A in terms of reasons for treatment failure. The prevalence of signature RAS at L31 and/or Y93 in NS5A in patients who stopped the therapy by adverse events was significantly low than others.

We then examined the association between reason for treatment failure and the prevalence of treatment-emergent RASs (Fig 4). The prevalence of RASs at positions L31 and/or Y93 was as follows: 91% in patients with virological breakthrough, 94% in patients with virological relapse after treatment, 100% in patients with primary non-response to therapy, and 78% in patients who prematurely discontinued therapy due to adverse events. The prevalence of signature RASs in L31 and/or Y93 in patients who prematurely discontinued therapy due to adverse events was significantly lower than others (p = 0.005). Similarly, the prevalence of dual RASs (L31-RAS/Y93-RAS) was significantly lower in patients who prematurely discontinued therapy due to adverse events than others (74% in breakthrough, 67% in relapse, 67% in non-response, and 22% in discontinued patients; p = 0.021).

### Prevalence of RASs other than L31 or Y93 in NS5A after treatment failure

**Fig 5:**
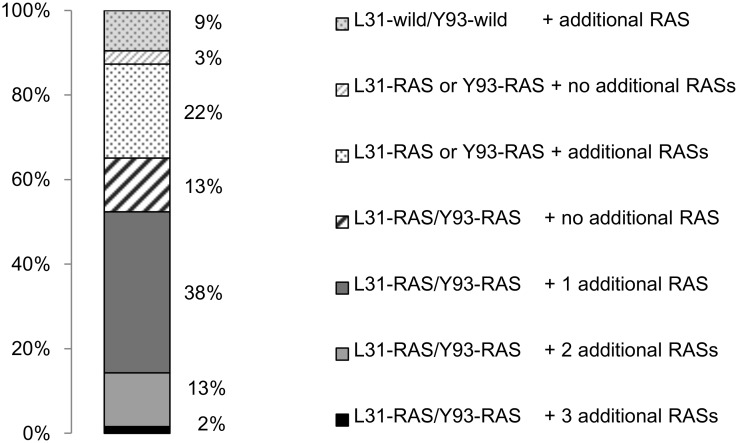
Prevalence of other RASs within NS5A in combination with signature L31/Y93 RAS. Other RAS in combination with dual signature RAS (L31-RAS/Y93-RAS) were detected in 53% of patients. The prevalences of triple, quadruple, and quintuple RAS were 38%, 13%, and 2%, respectively.

Of the 40 patients with dual signature RASs (L31-RAS/Y93-RAS), 5 patients had additional L28 RAS, 5 had additional R30 RAS, 1 had additional P32 RAS, 22 had additional Q54 RAS, 2 had additional P58 RAS, and none had additional A92 RAS. In total, other RASs in combination with dual signature RASs (L31-RAS/Y93-RAS) were detected in 53% of patients. The prevalence of triple, quadruple, and quintuple RAS was 38, 13, and 2%, respectively (Fig 5). All patients with L31-RAS/Y93-wild or L31-wild/Y93-wild and 71% of the patients with L31-wild/Y93-RAS had other RASs in NS5A region.

### Co-existence of NS5A and NS3 RASs after treatment failure

**Fig 6:**
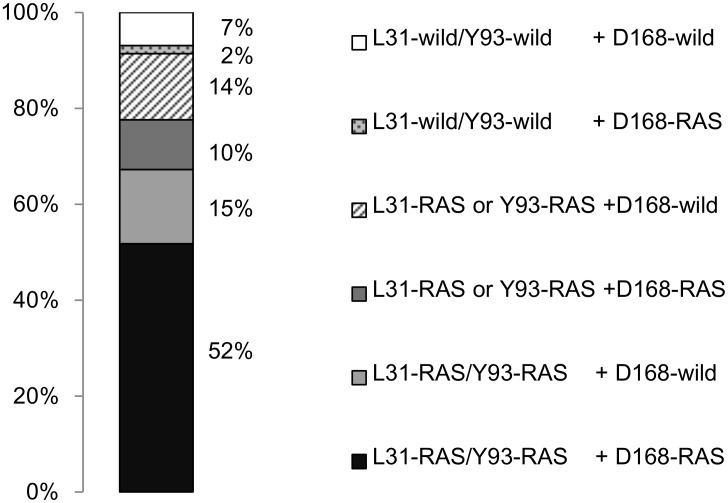
Co-existence of signature RASs in NS3 and NS5A. The prevalence of co-existence of signature RAS in NS3 and NS5A was analyzed. The prevalence of concomitant D168 RAS with L31 and/or Y93 RAS was 62%.

The prevalence of signature RAS at position D168 of NS3 was 68%. The prevalence of concomitant signature RAS in NS3 and NS5A was analyzed in 58 patients (Fig 6). The NS3 D168 RAS co-existed in 30 patients with dual RASs in NS5A (L31-RAS/Y93-RAS) and in 6 patients with single RAS in NS5A (5 patients with L31-RAS/Y93-wild and 1 patient with L31-wild/Y93-RAS). In total, co-existence of D168 RAS with L31 and/or Y93 RAS was observed in 62% of patients. Remaining 29% of the patients had NS5A RAS only, 2% had NS3 D168 RAS only, and 7% had no signature RAS in either NS3 or NS5A.

## Discussion

In this study, we have demonstrated the highly complex nature of RASs in NS5A region of HCV after treatment failure using DCV/ASV combination therapy in a sufficient number of patients. Signature RASs in L31 and/or Y93 were repeatedly reported [1,17,18]. However, this study revealed that the majority of patients with signature dual RASs (L31-RAS/Y93-RAS) carried additional RAS in the NS5A region, leading to a high prevalence of triple, quadruple, and quintuple RASs in 38, 13, and 2% of patients, respectively. These complex patterns of RASs were not associated with baseline factors, and were detected even in patients who prematurely discontinued therapy. The identification of these RASs, which may confer high levels of resistance to NS5A inhibitors, indicates the need for RAS determination when considering re-treatment with regimens that include NS5A inhibitors.

Currently, interferon-free combination of 2 or 3 classes of DAAs is the mainstay of treatment for genotype 1 chronic hepatitis C [1,24,25,23,31,32]. Of the three classes of DAA, NS5A inhibitors represent the key class included in the majority of combination therapy regimens. The presence of baseline RAS in NS5A does not attenuate the efficacy of the most recent treatment regimens in DAA-naïve patients [24,25,23]. However, evidence for the efficacy of these regimens in the re-treatment of patients who failed prior treatment with regimens that include NS5A inhibitors is lacking. Reduction in efficacy of retreatment with SOF/LDV after DCV/ASV failure was reported in one study with small number of patients [33]. This results should be validated by larger cohorts, but it may be obvious that efficacy of SOF/LDV is reduced in patients who failed prior NS5A inhibitor containing regimen. Therefore, current EASL guidelines do not recommend the use of NS5A inhibitors for re-treatment [34], whereas AASLD/IDSA [26] and JSH guidelines [27] recommend testing for RAS and do not recommend the use of NS5A inhibitors in patients found to have NS5A RAS. The key concept is that treatment-emergent NS5A RAS induced by treatment failure may confer higher levels of cross-resistance to NS5A inhibitors compared to naturally existing RAS. Our findings regarding the complex nature of RASs after treatment failure with the DCV/ASV combination regimen may support guidelines based on this concept.

The prevalence of naturally existing RASs in NS5A among DAA-naive patients has previously been reported [1,16,35], with the prevalence of Y93 and L31 RAS reported to be 20 and 4.5%, respectively in a cohort of 493 patients [16]. RAS at Y93 and L31 are associated with cross-resistance to several NS5A inhibitors [4,5,7–9,17–22,36], and dual RASs (L31-RAS/Y93-RAS) are associated with substantially higher levels of resistance [5,8]. However, the prevalence of dual RASs in DAA-naive patients is very low [16]. This study not only revealed a high prevalence (63%) of signature dual NS5A RASs (L31-RAS/Y93-RAS) in patients who failed prior therapy with DCV/ASV combination regimens containing NS5A inhibitors, which is in accordance with a previous report [1], but also depicted a high rate of co-existence of additional RAS in L28, R30, P32, Q42, and P58, leading to high prevalence of triple, quadruple, and quintuple RASs in combination with dual signature RASs. Unfortunately the presence of these RASs at baseline were not examined. Therefore we could not define whether these RASs were present at baseline or emerged after the therapy. The complex nature of RASs may confer high resistance to re-treatment as substantially enhanced resistance when combined with L31 and/or Y93 [5,8].

This study also revealed that a proportion of patients do not possess any RASs with presumed high levels of treatment resistance. Re-treatment using NS5A-containing regimens may have greater efficacy in these patients compared to patients with multiple RAS. Unfortunately, the presence of multiple RASs does not appear to be associated with baseline factors or reason for treatment failure. The signature RASs in L31 and/or Y93 were detected even in patients who discontinued therapy with short-term drug exposure, although the prevalence was lower than in patients with virological failure. Since adherence was 100% in all patients, the effect of reduced compliance on the appearance of RASs could not be evaluated in the present study. Moreover, treatment-emergent RASs in NS5A are reportedly replication fit and persist for long durations after treatment failure [18]. These observations indicate that testing for RASs provides important information regarding re-treatment, supporting the concept of current guidelines. Newer DAAs with high potency may provide better therapeutic effect in patients with identified RASs in the near future.

In conclusion, treatment-emergent RASs after treatment failure using DCV and ASV combination therapy are highly complex in more than 50% of patients. The identification of complex RAS patterns, which may indicate high levels of resistance to NS5A inhibitors, highlights the need for RAS sequencing when considering re-treatment with regimens that include NS5A inhibitors.
